# Volition or coercion?—the role of socio-psychological factors in the stratification pathways of Chinese university students

**DOI:** 10.3389/fpsyg.2026.1813219

**Published:** 2026-08-27

**Authors:** Keyu Zhai, Qingbin Xu, Meihan Tao, Weiyu Zeng, Yixin Zhao

**Affiliations:** 1 School of Education, Renmin University of China, Beijing, China; 2 Beijing Normal University, Beijing, China; 3 Tongling University, Tongling, China; 4 School of Graduate Studies, Lingnan University, Hong Kong, Hong Kong SAR, China; 5 Lingnan University, Hong Kong, Hong Kong SAR, China

**Keywords:** career attainment, Chinese university students, educational stratification, social stratification, social-psychological pathways

## Abstract

The existing literature on educational and social attainment has largely overlooked the role and interaction of social-psychological factors at the individual level. To address this gap, this study employs in-depth qualitative interviews (*N* = 45) to explore how such factors shape the academic and career trajectories of Chinese university students. Anchored in a social-psychological perspective, the research identifies six core thematic drivers: parental educational conceptions, individual educational and occupational aspirations, self-reflection practices, socially informed behavioral expectations, self-concept development, and peer influence dynamics. Building on these findings, the study develops a context-sensitive analytical framework that illustrates how established social-psychological mechanisms of status attainment operate within contemporary Chinese higher education system. Rather than proposing new social-psychological constructs, the study demonstrates how parental expectations, aspirations, self-concept, reflective practices, and peer influence interact with institutional features such as the Gaokao, rural–urban disparities, and changing labour-market conditions across different stages of educational and career attainment. In doing so, the study provides a qualitative refinement of the Wisconsin Model by specifying how its established mechanisms are configured within the contemporary Chinese context.

## 1 Introduction

A growing body of research has investigated education and social stratification (Buchmann and Hannum, 2001; Du, 2016; Posselt and Grodsky, 2017; Yeung, 2013; Zhai and Cao, 2025), because higher education reinforces social stratification, with pronounced educational inheritance and socioeconomic disparities (English and Umbach, 2016; Milkman et al., 2014; Mullen et al., 2003; Torche, 2011; Xie and Shauman, 2003). In particular, considerable scholarly attention has been devoted to developing countries such as China, which has been experiencing rapid higher education expansion and dramatic social changes (Mok et al., 2022). Researchers have found that although the expansion of higher education in China has increasingly generated education opportunities, students from disadvantaged socioeconomic backgrounds are more likely to be eliminated in the competition for elite higher education resources compared with their counterparts (Luo et al., 2018). However, the existing literature has largely overlooked the critical role of socio-psychological factors and individual-level agency within the stratification processes. Consequently, the subjective experiences, strategic decisions, and actual voices of students and their families have long been marginalized. There is, therefore, a timely imperative to develop a context-sensitive analytical application of the Wisconsin Model that better captures how established status-attainment mechanisms operate within contemporary China.

The existing literature categorizes the socioeconomic factors into three types of connotative capitals: economic, social, and cultural (Zhang and Wang, 2021). In terms of economic capital, the variable of family income level is widely discussed. High-income families have a positive impact on their children’s admission into higher educational levels (Luo et al., 2018; Dong and Chen, 2013; Tang, 2016; Ye, 2015). Social capital is linked to the social status, occupational status, political affiliation, which affects the educational attainments of their children (Zhang and Wang, 2021). Cultural capital affects educational attainments through factors such as the educational level of parents and the number of books in the family (Chunling, 2015; Hannum et al., 2011; Lai et al., 2016; Tang, 2016). Scholarly attention has been paid to the transmission effects of social and psychological factors, including parental expectations, school experiences, as well as students’ own educational expectations and visions (Mortimer and Lee, 2021; Jeffrey, 2020; Fishman, 2019). The insights from the macro-level research confirm that Chinese students from the disadvantaged family have certain drawbacks in access to higher education (Yip et al., 2020; Luo et al., 2021; Kohtamäki et al., 2024). The capital theories highlight the convertibility mechanism that higher education reproduces inequality by privileging cultural capital and elite networks. However, the literature should pay greater attention to individual socio-psychological factors in stratification pathways, because they are crucial mechanisms that translate structural opportunities into actual outcomes.

Aiming at bridging these gaps, this research would examine how structural inequalities intersect with cognitive factors in graduate admissions and labor market outcomes, thereby advancing a context-sensitive understanding of how established status attainment mechanisms operate within China’s distinctive higher education system while informing comparative research on educational stratification across different institutional settings. Methodologically and theoretically, this study collected the in-depth interview data and structured by the Wisconsin Model to conduct the qualitative data analysis. Rather than introducing new social-psychological mechanisms, this study examines how the established mechanisms identified in the Wisconsin Model are expressed and reconfigured within contemporary China. Specifically, it investigates how parental expectations, aspirations, self-concept, significant others, and behavioral expectations interact with institutional arrangements, including the Gaokao system, rural–urban inequalities, and rapidly changing labor markets, to shape educational and occupational trajectories. The contribution therefore lies in specifying the contextual configuration of status attainment processes.

Building on the Wisconsin Model, this study explores how established social-psychological mechanisms operate within China’s contemporary higher education system. Specifically, it seeks to examine (1) how family, institutional, and interpersonal factors shape educational and occupational trajectories; (2) how these mechanisms interact across different stages of status attainment within the Chinese context. Through qualitative interviews, the study provides a context-sensitive understanding of the operation of status attainment processes in contemporary China.

## 2 Literature review

### 2.1 From single to multiple factor theoretical system

The research on the interrelationship between education and social stratification has evolved from examining single objective factors toward a more comprehensive, multi-level analysis, supported by increasingly sophisticated theories and research methods. Since the 1990s, academic discussions of education and social stratification have been continued to evolve. In the late 20th century, research primarily focused on the impact of gender, distinguishing its effects on access to educational opportunities, experiences within higher education, and post-graduation outcomes (Jacobs, 1996; Davies and Guppy, 1997). After the 21st century, the influence of the previous generation, especially that of parents, has increasingly come under the spotlight. Some studies have revealed the influence of parental income on students’ enrollment rates and the impact of parents’ occupational status on their children’s future career positions (Conley, 2001; Korupp et al., 2002). In this initial phase, the research agenda was mainly concerned with the context of social change and upheaval, against which relevant theories and research content gradually expanded and diversified. Notably, in developed countries, population migrations have spurred reflections on the role of ethnic factors in educational stratification. Such research has been particularly prominent in the United States, where numerous scholars have identified that ethnic minorities, including Chinese Americans and African Americans, face disadvantages in accessing educational opportunities (Butler, 2004; Dickerson and Jacobs, 2006; Karen, 2002). Meanwhile, some research has shown that social changes in Nepal have led to a decline in women’s educational attainment (Beutel and Axinn, 2002). Besides, there have been increasingly extensive discussions about the decisive role of China’s household registration system in determining social stratification, manifested mainly in the impact of agricultural and non-agricultural household registrations on individuals’ employment, educational opportunities, and housing conditions (Wu and Treiman, 2004). In the early 21st century, academic exploration of relevant concepts remained at a superficial level, primarily focusing on observable, material-based variables, with relatively few studies delving into latent variables related to deeper- seated social-psychological aspects. The advantaged methods and more specific considerations in a context call for a multiple factor system to assess educational and social stratification. More importantly, the research on educational and social stratification increasingly calls for a framework that incorporates social-psychological mechanisms at the individual level.

### 2.2 Complex educational expectations

A growing body of literature began to emphasize the significance of educational expectations from the 2000s. Educational expectations among different ethnic groups of color in South Africa, revealed that, after controlling for other variables, the educational expectations of black individuals were higher than those of other ethnic groups (Beutel and Anderson, 2008). Additionally, it has been demonstrated that the educational expectations of American parents have been steadily increasing. Although family educational expectations are a direct predictor of educational outcomes, their connection with social backgrounds and career plans has gradually weakened (Faas et al., 2013; Goyette, 2008). Building on these findings, research interests have gravitated toward identifying the factors that influence educational expectations. More studies have started to explore the interplays of parental educational attainment, possession of a college degree, economic factors and moral values (Augustine, 2017; Bai, 2017; Chykina et al., 2016; Xu, 2016).

After 2020, studies have delved deeper into understanding how educational expectations influence education and social stratification. By introducing the perspective of absolute inequality, Jeffrey (2020) finds that students’ school experiences, shaped by their diverse backgrounds, can impact their personal expectations and capabilities, thereby influencing future social stratification. Moreover, research has indicated that, in addition to parental expectations, the socioeconomic composition of schools and associated norms are also significant factors influencing Hong Kong students’ educational expectations (Keung and Ho, 2020). Furthermore, academic discussions on educational expectations have evolved from static variable-based analyses of influencing factors to a more dynamic exploration of how educational and career expectations are formed at different life stages. In Germany, Dochow and Neumeyer (2021) investigate junior high school students’ educational expectations, which are shaped by personal cognition but have limited short-term impact on future achievements (Dochow and Neumeyer, 2021). Beyond personal cognitions, Mortimer and Lee (2021) employ the status-attainment theory and revealed that parents’ educational expectations can influence their children’s motivation and academic performance at school, ultimately then affecting future educational attainment and social stratification. In line with the research of Mortimer and Lee (2021), Kong (2024) highlights the equally important roles of fathers and mothers in shaping children’s early cognitive development and educational expectations.

Within the realm of educational sociology, pertinent research has evolved from a single factor to a more in-depth analysis of multiple factors. The extant research has not only identified a range of objective factors that impact individual educational attainment and social stratification but has also delved into analyzing how these factors shape the final outcomes. Notably, the status-attainment model has been frequently invoked in these studies, which may be attributed to its high degree of congruence with the research question at hand, namely, how parental-generation information influences children’s educational and career attainment. For developing countries, particularly China, during the processes of higher education development and social transformation, it is of utmost importance to conduct in-depth analyses of the underlying mechanisms of education and social stratification, such as exploring the influence pathways of social-psychological factors, which also enriches the existing theoretical framework.

## 3 Theoretical framework

**Figure 1:**
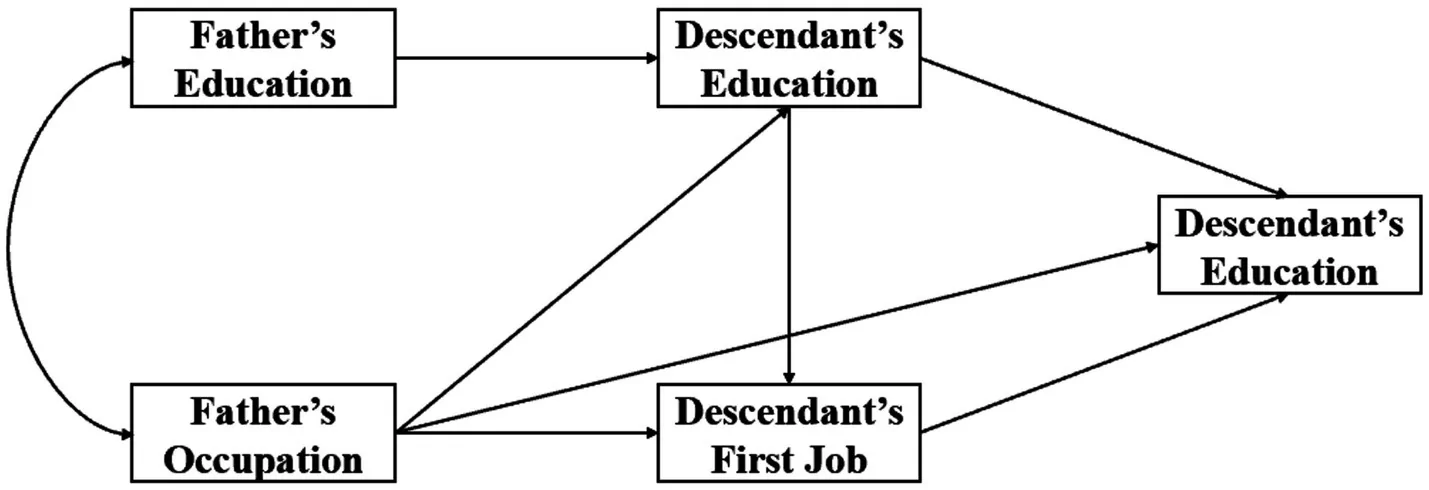
Classic status attainment model.

In the realm of educational sociology, the discourse surrounding status attainment theory unfolds as a rich exchange of ideas and refinements. The classic Status Attainment Model, as proposed by Blau and Duncan, posits a straightforward yet influential argument: a father’s educational attainment and occupational status serve as the primary architects of their offspring’s educational and occupational destinies, as illustrated in Figure 1.

However, as the scholarly conversation has evolved, the limitations of this traditional framework have become apparent. Critics, notably Fägerlind (1987), have argued that the model’s reliance on simplistic background variables, mainly confined to socioeconomic and cultural capital, failing to capture the full complexity of status attainment. Addressing this critique, the Wisconsin model emerges as a significant extension and refinement of the theoretical framework.

**Figure 2:**
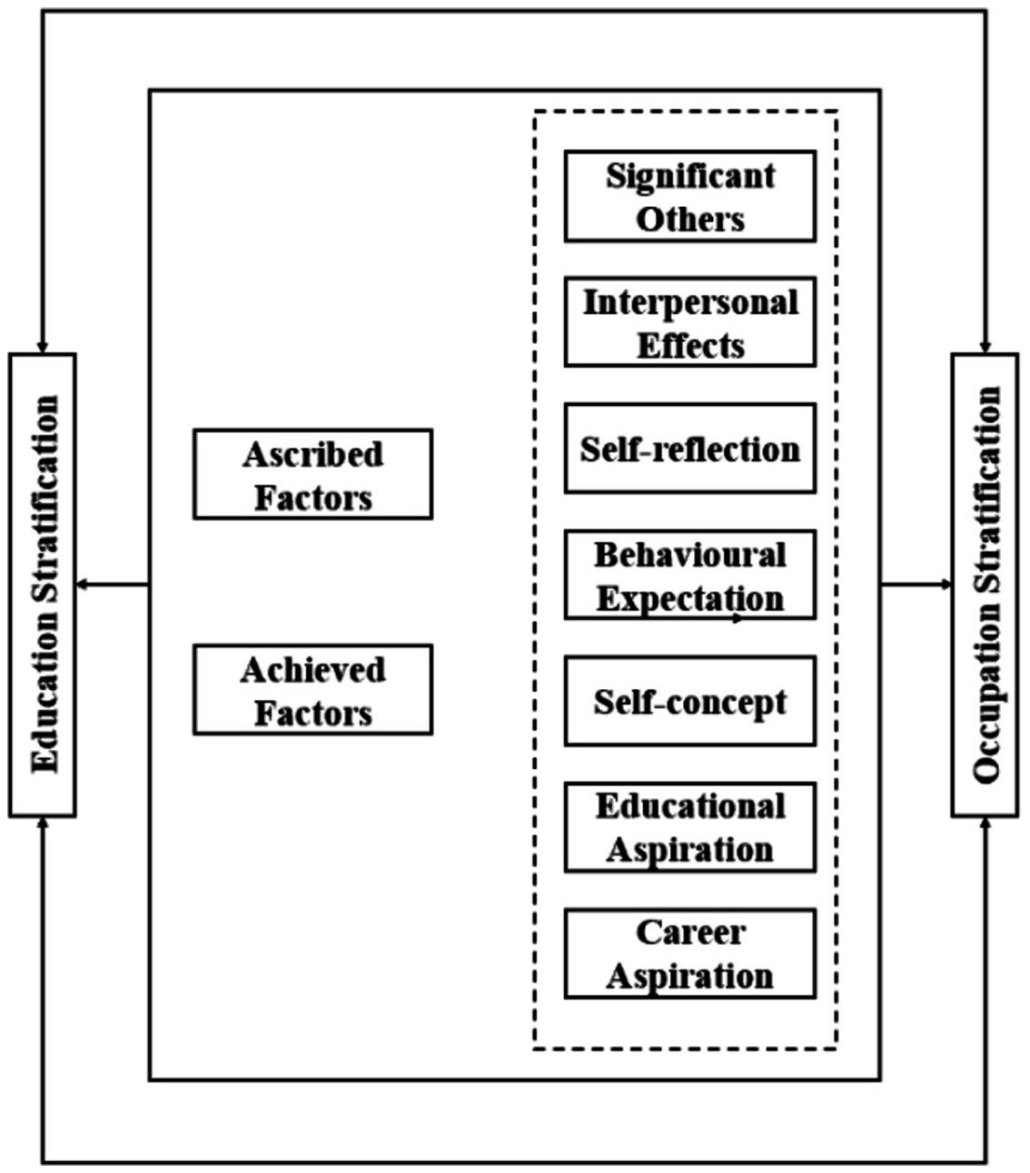
The Wisconsin model.

The proponents of the Wisconsin model, such as Bozick et al. (2010) and Scott and Marshall (2009), highlight the social and psychological factors in the status attainment process. Central to their framework is the concept of educational aspiration as a key mediator in intergenerational status transmission. This perspective is further enriched by scholarship examining the influence of significant others. Research by scholars such as Jeffrey (2020), Buchmann and Dalton (2002), Dyce et al. (2013), and Zuccotti et al. (2017) demonstrates how peers, friends, and teachers actively shape educational and occupational trajectories, as outlined in Figure 2.

By incorporating behavioral dimensions including self-concept and reflective practices, the recent research (Carvalhaes et al., 2023; Mortimer et al., 2017) enriches the mechanism through which individuals perceive themselves and reflect on their experiences. By introducing six additional dimensions, as illustrated by Sewell et al. (2018) and Sewell and Hauser (1975), the Wisconsin model not only addresses the limitations of the traditional model but also expands the theoretical toolkit available to researchers.

A crucial point of contention arises regarding the contextual applicability of these theories. Originally conceived within capitalist frameworks, their relevance to socialist settings like China is actively debated, necessitating a nuanced adaptation to distinct socio-institutional conditions. The Wisconsin Model has long emphasized social-psychological mechanisms such as parental expectations, aspirations, self-concept, and significant others in the status attainment process. Engaging with this theoretical dialog, this study seeks to refine the contextual application of the Wisconsin Model by identifying how its established mechanisms operate across different stages of educational and career attainment in contemporary China.

## 4 Methodology

### 4.1 Sampling and participants

Data were collected through in-depth interviews with 45 respondents, aged 20 to 35 years, from 21 provinces across China, to explore educational and occupational stratification. To establish a common baseline for analysis, all respondents had work experience and had entered higher education through the National College Entrance Examination (Gaokao). A purposive sampling strategy was used to ensure demographic and educational diversity, specifically encompassing graduates from the full spectrum of tertiary institutions—including junior colleges (专科), private institutions (民办), non-prestigious public universities (双非), elite national projects (Project 211 and 985 universities), C9 League institutions, and overseas universities. Notably, nearly half of the participants originated from rural areas, capturing the critical rural–urban dimension of social mobility in the Chinese context. This strategic composition of the sample was designed to align directly with the research objectives, thereby enhancing the validity and representativeness of the findings.

### 4.2 Data analysis

The interview protocol was informed by social constructivism (Berger and Luckmann, 2023), adopting its core premise that societal behavior emerges through cognitive processes mediated by subjective experience and reflexive interpretation. The framework’s axiomatic principle—“cognition constructs behavior”—informs our investigation of the behavioral translation of cognitive schemas during social construction. Braun and Clarke’s (2006) six-phase thematic analysis was adopted for data analysis, systematically coding interview data through a theoretically derived analytical framework (see Table 1). The interview protocol was explicitly designed to operationalize constructivist principles, enabling rigorous categorization of emergent themes while maintaining epistemological consistency with our theoretical foundations. Semi-structured interviews were guided by broad questions concerning participants’ family background, educational experiences, parental expectations, educational aspirations, career planning, peer influence and perceptions of social mobility. Follow-up questions were used to explore participants’ experiences in greater depth.

**Table 1:** Six steps of thematic analysis.

Steps	Description
Familiarization with the data	Analyze the interview data word by word
Generate initial codes	Code the words and sentences that meet the requirements
Explore themes	Assign themes to each of the primary codes
Review themes	Determine whether the existing themes are in line with the actual research
Define themes and name them	Adjust the original themes to better meet the needs
Write the report	Analyze each theme to draw discussions and conclusions

The study follows the well-defined thematic analysis approach proposed by Braun and Clarke, which involves a systematic and in-depth examination of the collected data. As outlined in Table 1, the thematic analysis process is structured into six sequential steps (Braun and Clarke, 2006).

As illustrated in Table 2, following the processes of primary coding, theme exploration, and theme review conducted using NVivo14 software, the data were categorized into six distinct themes in alignment with the Wisconsin theoretical model. Each of these themes was further subdivided into several sub-themes to facilitate a more granular analysis.

**Table 2:** Themes and sub-themes.

Themes	Sub-themes	Descriptions	Reference points
Influence of significant others	Influence of Parents	Parents have certain expectations or influences	179
	No Overt Parental Expectations	Parents have no actual expectations	18
	Importance of Family	The importance of the family in educational and occupational attainment	76
	Influence of Others besides Parents	The influence of other significant others besides parents	26
Educational and occupational aspirations	Personal Likes and Dislikes	Preferred educational and occupational directions	150
	Personal Efforts	Efforts made to achieve one’s vision	40
Self-reflection	Lack of Awareness	Lack of awareness in aspects such as educational or occupational choices	39
	Experiences of Others	The experiences of others make the subjects reflect on themselves	41
	Discrepancy between Reality and Ideal	The ideal state of the subjects does not match the actual situation	74
Self-concept	Personal Strengths	The directions of things that the subjects are good at	21
	Personal Weaknesses	The directions of things that the subjects are not good at	31
	Life Values	Whether the subjects’ cognition leans toward being positive or negative	92
Behavioral expectations	Market Situation	Taking actions based on the expected market situation	75
	Educational Environment	The expected influence of the educational environment on the results	68
Interpersonal influence	Influence on Willingness	Promoting or reducing the willingness in a certain direction	21
	Influence on Choices	Facilitating the choice in education and occupation	19

Following the initial inductive coding, the emerging themes were iteratively compared with the conceptual dimensions of the Wisconsin Model. The theoretical framework served as a sensitizing device to interpret how participants described educational and occupational decision-making. The final themes therefore reflect both empirical patterns identified through thematic analysis and their theoretical interpretation within the status attainment framework. This iterative process enabled the study to connect individual narratives with broader sociological explanations of educational and social stratification.

## 5 Findings

Thematic analysis revealed consistent patterns across ascribed backgrounds, with “influence of significant others” emerging as the most prevalent theme (299 references), particularly referring to parental influence. This was followed by “educational and occupational aspirations” (190 references), while “self-reflection,” “self-concept” and “behavioral expectations” each yielded approximately 150 references. “Interpersonal influence” appeared least frequently (50 references). Among these factors, the influence of significant others, especially that of parents, is pivotal, pervading the entire educational and career attainment process, in early educational attainment stage, higher education learning stage, and career attainment.

### 5.1 Influence of significant others

As “influence of significant others” includes crucial social connections like parents, elders, and teachers, some respondents indicated their parents, both urban and rural, generally only hoped them would enter colleges without specific school or major preferences. The reasons could be divided into 2 ways. On the one hand, some parents, lacking knowledge about universities and majors, only had basic expectations about college tiers, like institutions of 211 or 985 projects. On the other hand, others, despite having relevant information, believed that their children should not be overly interfered with. Some rural respondents mentioned that parents had limited knowledge of majors and no specific expectations, when they filled out college entrance examination application forms. For instance, a rural respondent stated:

*Since my parents are both agricultural-hukou holders, they do not know much. Anyway, their expectation is just for me to work hard on my own. They want me to develop in big cities. At that time, based on my high-school grades, it was no problem for me to get into a second-tier college.*

While urban and rural parents commonly aspire for their children to attend elite colleges, there are only differences in the nuances. Urban parents customarily invest in extracurricular tutoring for their children. Conversely, some rural parents solely endorse formal curricula and dismiss other educational modalities and focus on “gaining face” or “earning respect.” 2 rural respondents remarked:

*In my rural hometown, there is no concept of extracurricular activities or interest classes at all. For my mother, going to school is the only right path. Things like learning music, art, or doing sports are all considered improper.*

*My parents have relatively low educational backgrounds. As long as I can get into a good college, it will make them look good, and they do not have many other requirements.*

Parental expected educational directions are closely linked to future career paths and their experiences, and they cited “stability” as the top consideration, following with “high social status” and “high income.” Many, both urban and rural parents, strongly desire their children to enter the public sector. Additionally, girls are often directed toward teaching, and boys toward science and engineering. Furthermore, some parents, drawing on personal experiences, advise children to avoid certain industries. An urban respondent stated:

*I personally prefer pure science in the sciences, and my mother also likes research-oriented fields. She doesn’t particularly like applied fields. Among pure science disciplines, we ruled out chemistry first because both my parents work in the field of chemistry, and they think it’s harmful to the body and dangerous. Then I could choose from the remaining ones.*

Regarding selection of education or career location, the difference is mainly illustrated between gender. Many female respondents’ parents often advise their daughters not to choose universities or jobs located in remote cities. The reasons include convenience in life, safety, easy care, and concern for their children. After the early stage of higher education choices, urban and rural parents exhibit marked differences in their perspectives on children’s further education. Urban parents typically suggest further study options according to employment trends, whereas rural parents often prioritize economic considerations. Students in rural areas sometimes choose to pursue further education due to economic reasons. A rural respondent stated:

*My parents were concerned about economic factors. But I learned that if you get into graduate school, you’ll get a monthly stipend, and there are also scholarships if you perform well. I told my mother not to worry, saying that I would not ask them for money if I got into graduate school. And indeed, I got a first-class scholarship and never asked them for money. In fact, I even sent them money after getting the scholarship.*

Compared to parents’ influence on their children’s educational attainments, their impact on children’s career attainments is relatively less pronounced. When there is a substantial disparity between children’s and parents’ expectations, most children who are capable of independent living will not align with their parents’ suggestions but instead follow their own inclinations. Although students will choose their desired career paths when the conditions are opportune, some respondents admitted that their parents have a subtle influence on their educational and career attainment, as well as on their attitudes toward life and behavior:

*My parents’ influence on me is mainly subtle in two aspects. On the one hand, they hope I choose a more comfortable job and don’t have strong economic requirements. On the other hand, my parents don’t like to interact with other relatives and dislike socializing, which has led me to choose a major that seems to involve less social interaction and a region far from my current relatives.*

Therefore, the family background plays a significant role in educational and occupational attainments. From respondents’ statements, its importance can be distilled into two aspects: resource acquisition and spiritual support. Regarding resource acquisition, many respondents noted that with age and increased work experience, they gradually recognized family background’s crucial role in providing information sources. Respondents also elaborated on spiritual support, a factor that becomes especially conspicuous when comparing urban and rural areas. Urban parents typically offer a certain degree of spiritual encouragement, endowing their children with the fortitude to chase after their aspirations. Conversely, rural children often lack adequate spiritual sustenance, causing them to be more circumspect in their educational and career decision-making processes. For instance, a rural respondent stated:

*A better family background gives a person the courage to pursue many things. They have the confidence and will not hesitate. For example, if I wanted to take tutoring classes but my family didn’t have the money, I couldn’t. When I was in college, I wanted to take on challenges and pursue what I liked, but if my family background was better, I could freely pursue my interests.*

When examining “the influence of significant others” apart from parents, the influence mechanisms can be categorized into direct impact on respondents and indirect impact on respondents’ parents, with both positive and negative feedback. Parents might be swayed by others’ experiences to influence their children’s educational and career trajectories. For example, if a relative or friend has thrived in a particular industry, they may suggest their children select relevant direction. Conversely, others’ negative experiences can lead parents to deem a certain path unfeasible, generating negative feedback. The direct influence of others on respondents mainly originates from relatives, teachers, and friends. The following is an excerpt from an urban respondent who thought his relatives’ overseas study experiences shaped his educational choice:

*Regarding the choice of studying abroad, in my generation, for example, my cousins, both male and female, are all overseas students. Our family is at least not opposed to studying abroad. My parents even thought about sending me abroad before my college entrance examination. Thus, when choosing the overseas study program, I also thought it was a chance to get ahead.*

“The influence of significant others” largely originates from parental expectations. Even when some parents lack explicit expectations, they all fundamentally hope their children will succeed in education and career. These expectations powerfully drive children in their early years, shaping their cognition and interfering with their choices to some degree. However, as time elapses and children’s self-awareness grows, they will select their desired development paths. The influence of other significant individuals, such as relatives, teachers, and classmates, either impacts parental cognition or directly affects children’s decisions, with both positive and negative implications. Urban respondents are less constrained by parents materially and spiritually than rural counterparts. Urban parents typically possess broader knowledge and tend to let their children choose what they truly prefer. These narratives suggest that parental influence extends beyond the provision of material resources. Parents transmit cognitive frameworks regarding educational value, occupational desirability, and perceived opportunity structures, shaping how children interpret available choices long before making educational decisions. Consequently, structural inequalities are reproduced not only through unequal access to resources but also through unequal access to information, expectations and confidence.

### 5.2 Education and occupational aspirations

Among the respondents, a substantial proportion lack self-awareness and a clear career development trajectory, when facing the education choices, resulting in an unspecific plan when choosing an educational major. Excluding parental influence, most respondents choose the highest attainable educational level according to their individual circumstances. For Respondents with a certain level of self-awareness, it can be classified into multiple tiers. The first tier consists of those only aware of their dislikes. For instance, several respondents were clear about their aversion to mathematics, thereby demonstrating a preference in their major selection. The second tier pertains to being cognizant of one’s preferences or favored directions. For example, some respondents eschew jobs entailing interpersonal communication and, conversely, incline toward writing-related occupations. This has impelled them to select for the literature direction. The third tier involves having an extremely clear career direction. Consequently, these respondents initiate planning when choosing their educational routes, aiming to enter related industries in the future, like accounting or teaching. Between different family backgrounds, there is minimal variance in their early educational and occupational aspirations. These aspirations are all shaped by daily life, such as TV series, news and reading materials.

*In fact, I quite like the field of news dissemination. During my high school days, I also enjoyed watching interviews and reading magazines. That’s why at that time, and I had the intention of pursuing a career as a journalist.*

What substantially widens the gap between different family backgrounds and ascribed factors is the aspect of effort. Urban-background respondents are generally more proactive, whereas rural-background respondents are more passive in the effort-making process. Especially, the absence of guidance and assistance from significant others constrains their capacity to realize aspirations.

*It was the first time I left home. Coming to college marked my first departure from Guangxi and my arrival in a city in the north. I felt rather isolated and helpless. Moreover, my parents were unable to offer me substantial assistance. I realized that I had to forge ahead on my own path. Without the guidance of others, it was quite different from my high school days when I could receive help from my teachers or parents.*

These findings suggest that educational aspirations are shaped not only by individual interests but also by family support, available information, and perceived opportunities. Aspirations therefore reflect students’ interpretations of their social circumstances rather than purely personal preferences.

### 5.3 Self-reflection

Self-reflection emerges as a mechanism through which students reinterpret structural opportunities and constraints over time. As individuals accumulate educational and employment experiences, they revise earlier aspirations and behavioral expectations, illustrating the dynamic interaction between institutional conditions and individual agency. Theoretically, self-reflection pertains to an individual’s reflective conduct on past personal or others’ experiences, which influences subsequent educational or career choices. Several respondents indicated limited access to relevant information during past educational decision-making and confusion about future career development. They expressed a strong desire for professional guidance during educational choice-making, by both urban and rural respondents.

*Actually, I really wanted to pursue a technical direction, such as enrolling in a vocational and technical college for specialties like water conservancy and hydropower or railway. However, due to the lack of such information resources, I did not know that such options were available. If I had known, I think I would probably be working in the railway or hydropower field now.*

Except for insufficient information, the experiences of significant others also constitute a crucial dimension of educational decision-making. Typically, such reflection arises when respondents witness others’ educational or career choices resulting in either positive or negative outcomes. Subsequently, they engage in self-introspect and develop their own perspectives, some of which impact their personal decisions. Even when respondents make appropriate educational decisions, once they enter actual study or work, discrepancies with their expectations may emerge. For instance, after enrolling in a university major, they might find it does not lead to their desired industry, with a significant gap between the learned knowledge and their ideals.

Self-concept is shaped not only by personal abilities but also by continual social comparison and interaction with peers. Exposure to unequal educational and social resources influences how students evaluate their own capabilities and future possibilities, demonstrating how structural inequalities become internalized through everyday social experiences.

### 5.4 Behavioral expectations

The educational environment impacts on an individual’s behavioral expectations mainly occur prior to the National College Entrance Examination, before higher education choices. Educational environment can shape individuals. For example, key senior high schools, with their superior teaching atmospheres and resources, are more likely to foster higher score expectations, thus influencing educational attainment-related behaviors. However, some respondents argue that personal capabilities are more crucial than the educational environment, because they have relatively outstanding scores without a good education environment. Nevertheless, some respondents propose that scores do not fully represent educational achievements. Although quality-oriented education may not boost scores, its educational outcomes and comprehensive individual cultivation cannot be overlooked.

From an urban–rural perspective, these behavioral expectations are evident in the comparison before and after educational environment changes, reflecting the uneven distribution of educational resources. For example, respondents who transitioned from an urban to a rural educational environment experienced an academic performance deviation:

*I grew up in Ningbo. My parents sent me to a relatively good local school. Although the conditions were not very good, with the support of national policies and the implementation of compulsory education, there were basically no significant expenses. However, due to family reasons, I went back to my hometown to study in junior high school. My grades suddenly changed from being unremarkable in the class to becoming the first in the whole grade. But later, I gradually showed my true level, and my grades started to decline.*

The market situation primarily concerns how individuals, by aligning their future-development outlooks with self-awareness, shape or adjust their career-choice directions. Most respondents noted that the current job-hunting environment is highly competitive, presenting significant employment difficulties. As a result, they have modified their career choices. Some respondents, in response, have chosen to pursue further education, while others have changed their mindsets and targeted entry into the public sector, even though they had not previously intended to enter the public sector before labor-market conditions deteriorated. This finding is closely related to the self-reflection theme discussed above. These findings suggest that behavioral expectations represent adaptive responses to changing institutional environments rather than fixed personal preferences. Students continually adjust their educational and career decisions according to perceived labor-market conditions and educational opportunities, highlighting the reciprocal relationship between structural contexts and individual decision-making.

### 5.5 Self-concepts and interpersonal influence

“Self-concepts” pertain to an individual’s self-cognition, covering self-awareness of personal strengths and weaknesses, along with life values. Firstly, personal strengths are frequently reflected with personal preferences. For instance, if a student has consistently attained outstanding English grades in high school, they are likely to choose an educational path related to English majors. The formation of self-concept is closely linked to an individual’s experiences and lives. This indirectly explains the divergence in life values between urban and rural background individuals. This is exemplified by rural background students after entering higher education, because they may observe that classmates from affluent families possess resources that significantly support their studies and daily lives. Consequently, they may start to perceive personal efforts as negligible, losing their motivation to strive and the impetus to comprehensively plan for future development, which formulates a negative attitude toward life values.

*In our postgraduate class, there are many classmates who study extremely hard, or are extremely proficient in their majors, or possess great personal talents. However, due to factors such as their personal growth experiences or family backgrounds, they may choose to return to a small county town, earn a monthly salary of just a few thousand yuan, and engage in a job that has little to do with their majors. I think this is likely to be greatly influenced by their family environments. On the other hand, there are some classmates whose academic performance may not be outstanding and who have rather ordinary qualifications. But their parents, who are perhaps highly educated, will provide them with excellent career guidance.*

“Interpersonal influence” mainly concerns the impact on an individual’s educational attainment and career path via their interactions and associations with others. It can be classified into two sub-themes: the sub-theme of intention and the sub-theme of choices with a well-defined direction. The influence on intention typically during educational decision-making. For example, during the learning process, individuals often mutually encourage and support one another. In the context of choosing between liberal arts and science, some respondents may be influenced by their friends:

*Most of my friends are girls, and most of them are more proficient in liberal arts. Therefore, in fact, I also have a greater inclination towards choosing liberal arts. At that time, I got better grades in politics, history and geography, and I felt that I was more competent in these subjects. Besides, among my classmates, the few friends I was close to also chose liberal arts.*

The influence of choice occurs more frequently during the period of career attainment, and it is mainly manifested in the choices of the employment city and career direction. Many subjects have considered moving to cities where they have a large number of friends or classmates:

*I also stayed in Guangzhou after graduation because many of my university friends, and even high school friends and close friends, were employed in Guangzhou.*

*After 2020, the employment pressure became relatively high, so I gave up my original job. One of my university classmates had a good job in Chengdu, and he invited me, so I went to Chengdu to see if it was suitable for me there.*

These findings suggest that self-concept develops through continual interaction with educational experiences and social comparison. Exposure to different educational environments and peer groups shapes how students evaluate their own abilities and future possibilities, influencing subsequent educational and career choices.

## 6 Discussion

The findings illustrate how intergenerational expectations operate as a foundational mechanism in shaping early educational outcomes and cognitive dispositions, thereby contributing to the reproduction of inequality. During the formative years, parents act as primary agents of socialization and resource allocation. At this developmental stage, children’s developing sense of agency makes them particularly receptive to parental expectations, which are themselves informed by the parents’ own experiential and socio-cultural backgrounds. These expectations are often manifested through deliberate, goal-oriented support for educational and occupational advancement, namely, a process reflective of intentional intergenerational cultivation. While parental expectations, aspirations, and self-concept are widely recognized components of status attainment processes, the present findings suggest that their operation is closely conditioned by China’s institutional environment. Not functioning independently of context, these mechanisms are embedded within a highly examination-oriented education system, pronounced rural–urban inequalities, and rapidly changing labor-market conditions. For example, parental expectations are strongly oriented toward educational credentials because the Gaokao represents a high-stakes gateway to subsequent educational and occupational opportunities. Likewise, rural respondents’ aspirations are constrained not only by family resources but also by unequal access to educational information and institutional support, while increasing labor-market uncertainty encourages students to continually revise their educational and career expectations. These findings suggest that institutional arrangements shape not the existence of status attainment mechanisms, but their relative importance, sequencing, and interaction throughout the life course.

While urban and rural disparities in material and institutional resources are significant, a more fundamental distinction often lies in the cognitive frameworks parents hold regarding education, which are shaped by the breadth and nature of their social and economic exposure. For instance, some rural parents with extensive non-local or non-agrarian experience develop educational outlooks comparable to those of urban parents. In contrast, parents with more circumscribed social horizons may struggle to fully apprehend the long-term returns of schooling, resulting in a consequential gap in educational expectations. Beyond overt parental aspirations, daily behaviors, such as habitual reading or engagement with learning, serve as powerful implicit models that socialize children toward particular attitudes about education. This dynamic helps explain how certain families, even with limited economic capital, can effectively support high academic attainment through a consistent alignment of cognitive outlook and supportive practice, highlighting the interdependent roles of belief, behavior, and context in the process of intergenerational mobility. The transition to higher education marks a critical juncture where intergenerational influence shifts from direct transmission to mediated negotiation. Parental expectations, while enduring, are increasingly filtered through a rapidly expanding field of institutional and peer-based socialization. This exposure catalyzes the development of reflexive self-awareness, enabling students to consciously construct personal cognitions that may contest inherited scripts. For individuals from rural or otherwise constrained backgrounds, this process is often defined by comparative reflexivity, which is a heightened awareness of structural disadvantage relative to more privileged peers. This awareness generates a distinct psychosocial calculus, wherein ambitions are tempered by familial responsibility and perceptions of limited opportunity. The outcome is not merely a weakened parental imprint, but the active formation of a contextually embedded self-concept, synthesizing aspirational drives with a pragmatic navigation of social position. The career trajectories reveal a dynamic process of self-concept formation and behavioral adaptation shaped by market conditions. Respondents demonstrate cognitive flexibility, continually recalibrating their professional self-perceptions through experiential learning and reflective practices. For example, individuals who initially rejected institutional employment often recalibrated their preferences following challenging work experiences. This iterative self-correction mechanism operates through continuous feedback between self-reflection and behavioral expectations. Notably, while urban–rural disparities have been mitigated in occupational attainment, the salience of social capital increases, with interpersonal networks emerging as critical determinants of career progression.

Rather than operating independently, the identified socio-psychological factors form an interconnected process through which structural inequalities are gradually internalized into individual cognition and behavior. Family socioeconomic background influences parental educational expectations, which shape children’ aspirations and self-concept during the early stages of educational decision-making. These dispositions are subsequently reinforced or modified through institutional experiences, including the Gaokao system, unequal educational opportunities, and interactions with peers and teachers. As students progress into higher education and the labor market, reflexive evaluation of institutional constraints and employment opportunities reshapes their behavioral expectations and career decisions. Agency therefore emerges not as an alternative to structure but as a socially embedded process through which individuals interpret, negotiate, and respond to structural opportunities and constraints. This dynamic interaction helps explain how educational and social inequalities are both reproduced and, in some cases, renegotiated across different stages of status attainment.

**Figure 3:**
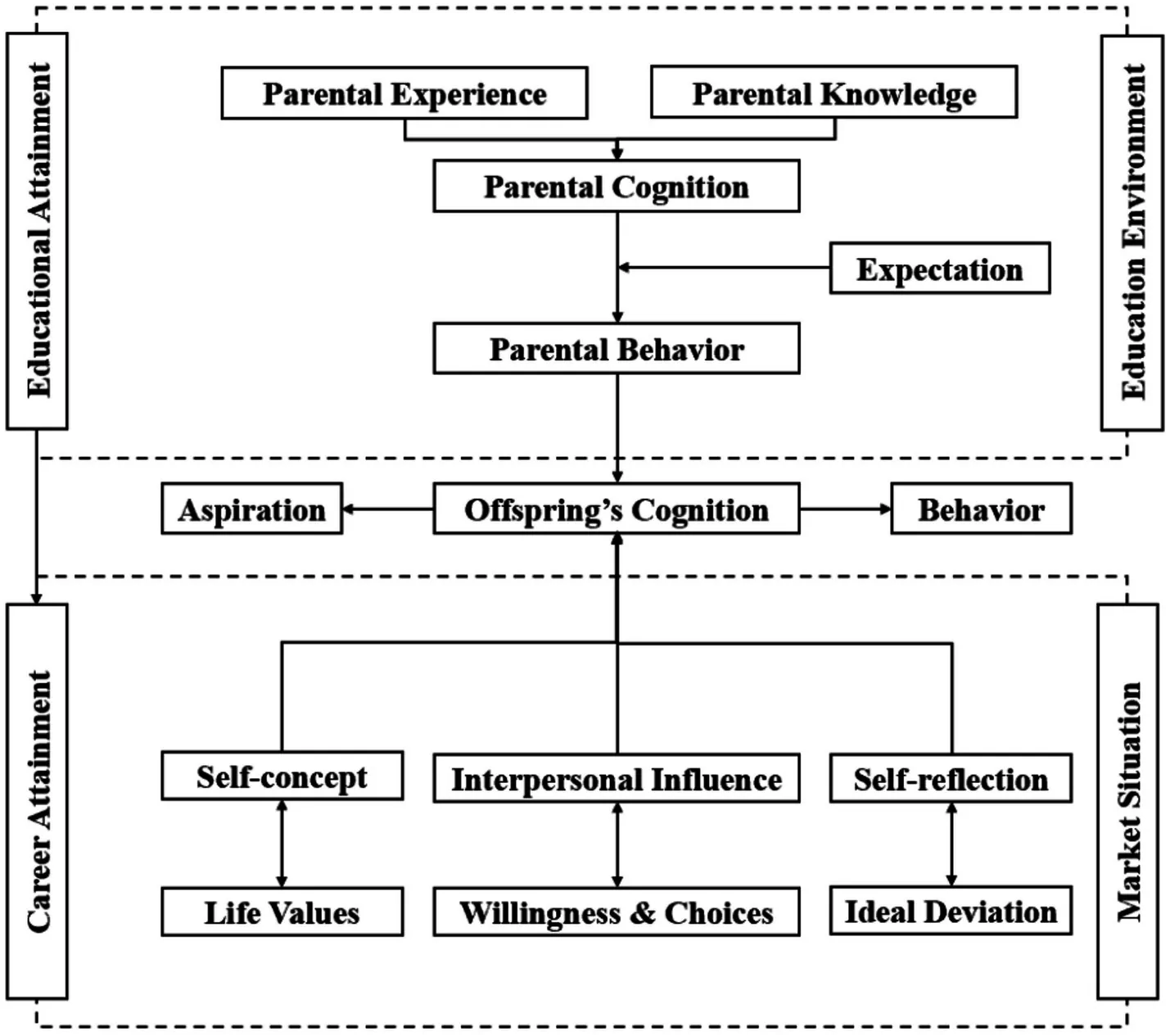
Context-specific status attainment pathways in contemporary China.

Building on the Wisconsin model, social constructivism, and the empirical findings, we present a context-specific analytical representation illustrating how established status-attainment mechanisms interact within contemporary China’s institutional setting. As depicted in Figure 3, the arrows denote the direction of construction. The upper part represents the educational attainment process, where parental cognition consisting of parental experience and knowledge, mainly manifested in the level of parental expectations, plays an important role. When students enter the labor market after graduation and start working in the lower part, their self-cognition is no longer solely constructed by parental cognition. Instead, they form their own understanding through self-concept, interpersonal communication, and self-reflection.

Throughout the construction of the social-psychological pathway, urban–rural differences are primarily reflected in the educational attainment process. Varying parental experiences and knowledge lead to different cognitions, which is the key to the disparities in educational attainment between urban and rural areas within this pathway. Moreover, when rural children enter a new collective, aside from educational attainment differences, psychological deviations may occur. In response, during the promotion of rural education, efforts should not only focus on increasing educational quantity and popularization but also pay more attention to students’ mental health education (Liu et al., 2021). Schools need to invest more in building relevant mental health teaching staff, and families should enhance parent training, conduct related activities, and strengthen quality education. Another crucial aspect is the popularization of occupations and majors, as this can widen the urban–rural gap. Rural parents often feel confused when their children choose majors and occupations, and this also happens in some urban families. Thus, it is urgent to establish a comprehensive career planning system. Professional teachers, college admissions officers, and workplace professionals should be invited to give regular lectures, along with case-based explanations and career evaluations, to help students understand the actual prospects of majors and occupations. Meanwhile, parents should be encouraged to participate in major choosing, actively learn relevant knowledge, and cultivate positive and healthy concepts. Relevant policies should also support the construction of vocational recruitment platforms and professional search platforms to assist rural students in screening suitable educational and career attainment directions.

## 7 Conclusion

This research addresses the limited research on the specific paths through which social-psychological factors influence individuals’ educational and career achievements. The findings suggest that, among the participants in this study, educational stratification was shaped by parental behavior and parental cognition, particularly through parental expectations. They jointly shape children’s cognition and thus influence their expectations and behaviors. Conversely, in the stage of career attainment, namely, the stage of social stratification, children mainly rely on their own cognition. When choosing the level and direction of their careers, they base their decisions on their self-concept and self-reflection, and parental behavior has less influence because they have a higher sense of autonomy compared to the previous stage of educational attainment. In the entire process of higher education stratification and social stratification, both ascribed and achieved factors will affect individual judgment. In the early stage, ascribed factors play a stronger role and its process is more affected by the educational environment. After entering society, achieved factors have a more significant effect, accompanied by the expected effect of the market environment. This study contributes to the Wisconsin Model by demonstrating how its established social-psychological mechanisms are conditioned by China’s institutional context. Rather than proposing a new status-attainment model, the study provides a context-sensitive analytical representation that illustrates how parental expectations, aspirations, self-concept, self-reflection, behavioral expectations, and interpersonal influence interact with institutional arrangements such as the Gaokao system, rural–urban inequalities, and changing labor-market conditions. In doing so, the study refines the contextual application of the Wisconsin Model and contributes to comparative research on educational stratification across different institutional settings.

This study yields significant policy implications by examining how socio-psychological factors shape decision-making among micro-groups navigating China’s educational stratification system. These cognitive mechanisms indirectly influence educational participation rates and ultimately challenge national goals of educational modernization. For example, when parents develop negative perceptions of education’s value based on limited experiences, such orientations become intergenerationally transmitted, creating psychological barriers that restrict offspring’s access to tertiary education. The research further reveals widespread uncertainty in educational/career decision-making across socioeconomic groups, often causing early aspirations to diverge from students’ actual needs and potentials. To address this, a targeted policy response should intervene at three critical junctures: establishing institutional career guidance to clarify education-to-work pathways, launching parental education programs to broaden perceptions of educational value, and creating adaptive university major-selection systems to better align with student aptitudes. This integrated approach would mitigate the intergenerational reproduction of educational inequality while improving the efficiency of human capital allocation.

## Data Availability

The original contributions presented in the study are included in the article/supplementary material, further inquiries can be directed to the corresponding author.
